# Reciprocal t(9;22) ABL/BCR Fusion Proteins: Leukemogenic Potential and Effects on B Cell Commitment

**DOI:** 10.1371/journal.pone.0007661

**Published:** 2009-10-30

**Authors:** Xiaomin Zheng, Claudia Oancea, Reinhard Henschler, Malcolm A. S. Moore, Martin Ruthardt

**Affiliations:** 1 Department of Hematology, Laboratory for Tumor Stem Cell Biology, Goethe University, Frankfurt, Germany; 2 Cell Biology Program, Memorial Sloan-Kettering Cancer Center, New York, New York, United States of America; 3 Department of Transfusion Medicine and Immunohematology, Goethe University, Frankfurt, Germany; KU Leuven, Belgium

## Abstract

**Background:**

t(9;22) is a balanced translocation, and the chromosome 22 breakpoints (Philadelphia chromosome – Ph^+^) determine formation of different fusion genes that are associated with either Ph^+^ acute lymphatic leukemia (Ph^+^ ALL) or chronic myeloid leukemia (CML). The “minor” breakpoint in Ph^+^ ALL encodes p185*^BCR/ABL^* from der22 and p96*^ABL/BCR^* from der9. The “major” breakpoint in CML encodes p210*^BCR/ABL^* and p40*^ABL/BCR^*. Herein, we investigated the leukemogenic potential of the der9-associated p96*^ABL/BCR^* and p40*^ABL/BCR^* fusion proteins and their roles in the lineage commitment of hematopoietic stem cells in comparison to BCR/ABL.

**Methodology:**

All t(9;22) derived proteins were retrovirally expressed in murine hematopoietic stem cells (SL cells) and human umbilical cord blood cells (UCBC). Stem cell potential was determined by replating efficiency, colony forming - spleen and competitive repopulating assays. The leukemic potential of the ABL/BCR fusion proteins was assessed by in a transduction/transplantation model. Effects on the lineage commitment and differentiation were investigated by culturing the cells under conditions driving either myeloid or lymphoid commitment. Expression of key factors of the B-cell differentiation and components of the preB-cell receptor were determined by qRT-PCR.

**Principal Findings:**

Both p96*^ABL/BCR^* and p40*^ABL/BCR^* increased proliferation of early progenitors and the short term stem cell capacity of SL-cells and exhibited own leukemogenic potential. Interestingly, BCR/ABL gave origin exclusively to a myeloid phenotype independently from the culture conditions whereas p96*^ABL/BCR^* and to a minor extent p40*^ABL/BCR^* forced the B-cell commitment of SL-cells and UCBC.

**Conclusions/Significance:**

Our here presented data establish the reciprocal ABL/BCR fusion proteins as second oncogenes encoded by the t(9;22) in addition to BCR/ABL and suggest that ABL/BCR contribute to the determination of the leukemic phenotype through their influence on the lineage commitment.

## Introduction

t(9;22)(q34;q11) is detected in 95% of chronic myeloid leukemia (CML) cases as well as in 20–30% of adult acute lymphatic leukemia (ALL) cases. CML is a myeloproliferative syndrome characterized by an indolent chronic phase (CP) with an overgrowing mature myeloid cell population, which is, if not treated, inevitably followed by an acute phase, the so-called blast crisis (BC). Clinically, BC resembles acute leukemia, with a poor prognosis and resistance to therapy [1]–[3]. CML-BC displays a myeloid phenotype in two-thirds of cases and a lymphatic phenotype in the remaining one-third [1]. In contrast, Ph^+^-ALL is an acute disease from onset and is characterized by blasts that are blocked, in the majority of cases, at the pre-lymphatic stage of differentiation. Patients suffering from Ph^+^ ALL constitute a high risk group of ALL [4]. The factors that determine the evolution of CML, as well as the biological differences between CML and Ph^+^-ALL, are almost completely unknown.

t(9;22) is usually a reciprocal translocation. A portion of chromosome 9 fuses to chromosome 22 (der22), thereby replacing a fragment of chromosome 22, which in turn fuses to chromosome 9 (der9). The cytogenetic correlate of der22 is the so-called Philadelphia chromosome (Ph). On chromosome 22, t(9;22) involves the *bcr* (breakpoint cluster region) gene locus. Two principal breaks occur: the (major) M-bcr, between exons 12 and 16, and the (minor) m-bcr, in the first intron of *bcr*. The breakpoint on chromosome 9 is consistently located in intron 1 of the *abl* gene locus. On der22, M-bcr leads to the creation of p210*^BCR/ABL^*, which is the hallmark of CML [1]. The Ph^+^ ALL-related p185*^BCR/ABL^* transcript is constant because the m-bcr breakpoint maps within an intron [1]. Although m-BCR (p185*^BCR/ABL^*-positive) is sporadically found in CML, it is considered to be specific for Ph^+^ ALL [5]. The *abl/bcr* fusion genes on der9 mainly differ in the specific breakpoint on chromosome 22. M-bcr results in the “small” *abl/bcr*, which encodes a “small” ABL/BCR transcript that is detectable in 65% of patients suffering from CML [6]. The resulting ABL/BCR fusion protein has a theoretical molecular mass of 40 kDa - p40*^ABL/BCR^*. The fusion between m-bcr and abl leads to a “large” transcript, which is present in 100% of examined patients with m-BCR Ph+ ALL [7]. This gene encodes a fusion protein with a theoretical molecular mass of 96 kDa - p96*^ABL/BCR^*.

The BCR/ABL fusion proteins are mutant ABL kinases. In normal cells, ABL kinase activity is finely regulated in response to growth factors and other stimuli. Through fusion to BCR, ABL becomes constitutively activated. This leads to constitutive activation of “down-stream” signal transduction pathways, including Ras, Jak/Stat and PI-3 kinase [1], [8]. The suppression of constitutively active ABL kinase with specific kinase inhibitors, such as Imatinib, Nilotinib [9] and Dasatinib [10], reverts the oncogenic potential of BCR/ABL.

The ABL/BCR fusion proteins are BCR mutants [11]. It has recently been reported that BCR is a negative regulator of proliferation and oncogenic transformation [12]. It associates with AF6 and Ras to form a trimeric complex, which is involved in the down-regulation of Ras-mediated signaling at sites of cell-cell contact to maintain cells in a non-proliferating state. The binding site for AF6 is located at the C-terminus of BCR [12]. Furthermore, BCR inhibits Wnt signaling by blocking Tcf-1/β-catenin-mediated transcription. It disrupts the Tcf-1/β-catenin complex by direct binding to β-catenin and Tcf-1 [13]. Through its Rho-GEF and Rac-GAP functions, BCR plays an important role in cytoskeleton modeling by regulating the activity of Rho-like GTPases, such as Rac, cdc42 and Rho [11], [14].

**Figure 1:**
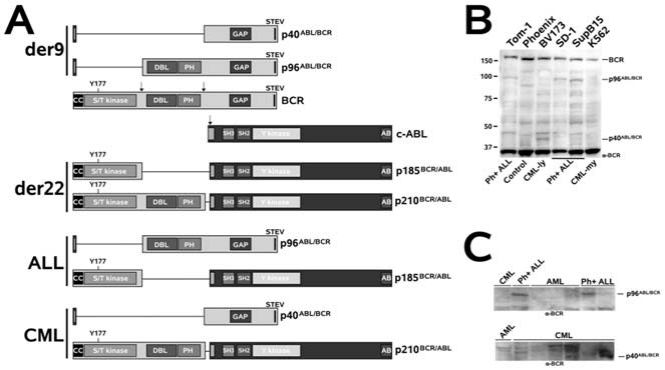
The t(9;22) fusion proteins and their expression in Ph+ cells. (A) Modular organization of the translocation partners and the fusion proteins in t(9;22). A schematic representation of the fusion proteins encoded by derivative 9 (der9) and 22 (der22), the Philadelphia chromosome, as well as of their combination in m-BCR-positive Ph+ ALL and M-BCR-positive CML, respectively. CC - coiled coil oligomerization interface; Y177 - Tyrosine phosphorylation site at aa 177; S/T kinase - serine/threonine kinase domain; DH - dbl homology domain; PH - pleckstrin homology domain; GAP - Rac-GAP domain; STEV - PDZ-domain binding motif; SH2 and SH3 - Src homology domains 2 and 3; Y kinase - tyrosine kinase domain; AB - actin binding domain. (B) Expression of the reciprocal ABL/BCR fusion proteins in Ph+ leukemia cell lines. Negative control: Phoenix cells; Tom-1, SD-1 and SupB15 - cell lines derived from m-BCR-positive ALL patient samples; BV173 - cells derived from a sample obtained from a CML patient in lymphatic BC; K562 - cells derived from a sample obtained from a CML patient in myeloid BC. (C) Expression of the reciprocal ABL/BCR fusion proteins in samples from Ph+ leukemia patients. Negative controls - three samples from AML patients and one sample from a CML patient for p96*^ABL/BCR^* in Ph+ ALL samples; one sample from an AML patient for p40*^ABL/BCR^* in CML samples.

Very little is known about the biology of p40*^ABL/BCR^* and p96*^ABL/BCR^* and their roles in leukemogenesis. In both ABL/BCRs, fundamental functional features of wt BCR, including regulation of small Rho-like GTPases, are lost with negative consequences on cell motility [11]. CML-associated p40*^ABL/BCR^* lacks the oncogenic DH/PH domains that are conserved in the ALL-specific p96*^ABL/BCR^* (Figure 1A). Thus, the ALL-specific p96*^ABL/BCR^* fusion protein is an N-terminally truncated Rho-GEF and, therefore, a putative oncogene [15].

Herein, we investigated the leukemogenic potential of the ABL/BCR proteins, as well as their influence on the lineage commitment of early hematopoietic stem cells.

## Results

### The reciprocal t(9;22) fusion proteins p40*^ABL/BCR^* and p96*^ABL/BCR^* are expressed in patient-derived Ph+ ALL and CML cells

The fact that all m-BCR-positive Ph+ ALL and about two-thirds of M-BCR-positive CML patients express the reciprocal fusion transcript strongly suggests a fundamental role of the reciprocal ABL/BCR fusion gene in induction of the leukemogenic phenotype. The modular organization of the translocation partners and the resulting fusion proteins from m-BCR and M-BCR in t(9;22), which are present in Ph^+^ ALL and CML, is presented in Figure 1A.

To confirm that the ABL/BCR transcript is translated into a functional protein, we first investigated the expression of p40*^ABL/BCR^* and p96*^ABL/BCR^* at the protein-level by Western blotting of extracts obtained from patient-derived Ph+-ALL and CML cells. We used SupB15, SD-1 and TOM-1 cells, as well as samples from 3 Ph+ ALL patients, all known to present m-BCR and expected to express the reciprocal p96*^ABL/BCR^*. The CML-blast crisis-derived BV173 and K562 cells and samples from 6 CML patients harboring the M-BCR, and thus expected to express the reciprocal p40*^ABL/BCR^*, were also included. Samples from patients with AML were used as negative controls. As shown in Figure 1B, all Ph^+^ ALL cell lines and 3/3 Ph^+^ ALL patients exhibited a band of about 96 kDa, which was absent in the CML-derived cell lines and in the negative controls. In contrast, a band of about 40 kDa was detected in only the lymphatic BV173 and in 3/6 CML patients. The myeloid K562 cells did not express the p40*^ABL/BCR^* (Figure 1B).

Taken together, these data show that the reciprocal ABL/BCR proteins were expressed not only at the transcriptional level, but also at the protein level in patient-derived cells. It is noteworthy that, in contrast to CML, all tested m-BCR-positive Ph^+^ ALL cells expressed the reciprocal p96*^ABL/BCR^*, suggesting a putative role for this fusion protein in induction of the leukemic phenotype.

### Both p40*^ABL/BCR^* and p96*^ABL/BCR^*, but not p185*^BCR/ABL^*, increase the replating efficiency of murine HSCs and block their myeloid differentiation

**Figure 2:**
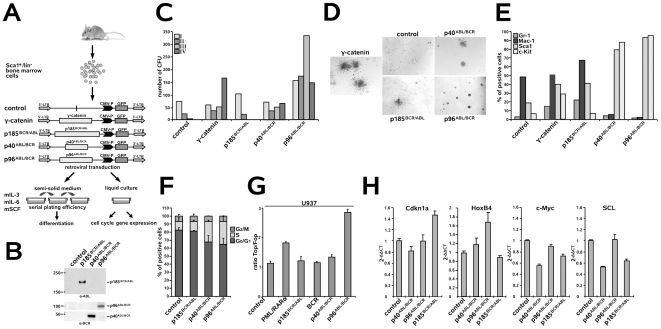
Effect of the t(9;22) fusion proteins on the biology of murine HSCs. (A) Experimental strategy for studying the influence of the t(9;22) fusion proteins on the biology of murine HSCs. Sca1^+^/lin^−^ BM cells were infected with the indicated retroviruses and plated in semi-solid medium supplemented with the indicated growth factors for determination of the serial replating potential. Cells from the first plating (I) round were examined for the expression of differentiation-specific surface markers. Cells plated in liquid culture supplemented with the indicated growth factors were used for cell cycle analysis and gene expression by qRT-PCR. (B) Sca1^+^/lin^−^ BM cells were infected with the indicated retroviruses and expression levels of the transgenes were analyzed by Western blotting using the indicated antibodies. (C) Long-term serial replating. Sca1^+^/lin^−^ cells were infected with the indicated retroviruses and plated into methyl-cellulose supplemented with the indicated growth factors to assess primary colony formation. Colony numbers were counted on days 8–10. Cells were then harvested and serially replated. Colonies were counted on days 8–10 after each replating. (D) Colony morphology during the first plating. Type A (compact colonies), type B (dense center surrounded by a halo of migrating cells) and type C (diffuse colonies with mobile differentiating cells) colonies were distinguished. (E) Expression of differentiation-specific surface markers. One representative experiment of three yielding similar results. (F) Sca1^+^/lin^−^ BM cells were infected with the indicated retroviruses and, after 48 h, cell cycle progression was determined. The provided results are the average of three independent experiments +/− SD. (G) Activation of Wnt-signaling by the t(9;22) fusion proteins. BCR, p185*^BCR/ABL^*, p40*^ABL/BCR^* and p96*^ABL/BCR^* and the Topflash and Fopflash reporter constructs were co-transfected by electroporation into U937 cells. U937 cells expressing PML/RARα - positive control; pGL3basic - negative control. Luciferase activity measured at 24 h post-transfection was normalized to Renilla luciferase activity. Each experiment was performed in triplicate a total of three times with similar results. One representative experiment is given +/− SD. (H) Sca1^+^/lin^−^ BM cells were infected with the indicated retroviruses and, at 48 h post-infection, the expression levels of HoxB4, Cdkn1a (p21^(cip1/waf1)^), c-Myc and SCL were analyzed by qRT-PCR. The relative concentration of each mRNA was normalized to the concentration of the housekeeping gene GAPDH and is represented as 2^−Δ/Δ^ CT. Each experiment was performed in triplicate a total of three times with similar results. One representative experiment is given +/− SD.

Both CML and Ph^+^ ALL have been proposed to originate from a transformed stem cell. Therefore, we compared the effects of the ABL/BCR fusion proteins and p185*^BCR/ABL^* on the biology of HSC. First, the *in vitro* replating efficiency in semi-solid medium in the presence of hematopoietic growth factors of Sca1^+^/lin^−^ HSCs transduced with p40*^ABL/BCR^*, p96*^ABL/BCR^* or p185*^BCR/ABL^* was determined. γ-catenin was used as a positive control [16](Figure 2A). Transgene expression was controlled in the colony forming units (CFUs) from the first plating (Figure 2B). Here, we show that the replating efficiency of the empty-vector transduced controls was limited to one cycle of replating (Figure 2C). In contrast, both p40*^ABL/BCR^* and p96*^ABL/BCR^* increased the replating efficiency over four cycles, with a maintenance or increase in the number of CFUs in each round of replating (Figure 2C). The morphologies of the colonies were classified as type A, B or C colonies according to the descriptions provided by Lavau and co-workers [17], [18]. As shown in Figure 2D, expression of p96*^ABL/BCR^* led to type A colonies, which are typical compact colonies, representing >90% of the p96*^ABL/BCR^*-positive colonies. Also, p40*^ABL/BCR^* gave rise to more than 80% type A colonies. In contrast, γ-catenin CFU colonies were mainly of type B, with a dense center surrounded by a halo of migrating cells, whereas empty-vector controls and p185*^BCR/ABL^*-positive cells exhibited the type C morphology of diffuse colonies with mobile differentiating cells (Figure 2D). The immature character of p40*^ABL/BCR^*- and p96*^ABL/BCR^*-positive CFU was confirmed by the high expression levels of c-Kit and Sca1, and low levels of the myeloid differentiation markers Gr-1 and Mac-1 in comparison to p185*^BCR/ABL^*-positive CFU and mock-transduced controls (Figure 2E).

To study the influence of the t(9;22) fusion proteins on the cell cycle progression of murine HSC, we maintained the transduced cells in liquid culture for 48 h prior to cell cycle analysis (Figure 2A). As shown in Figure 2F, expression of p185*^BCR/ABL^* did not lead to an increase in the percent of cells in S phase, whereas expression of either p40*^ABL/BCR^* or p96*^ABL/BCR^* led to a reduction in the percent of cells in G_0_/G_1_ phase with a proportional increase in the amount of cells in the S phase, as compared to the controls.

To explain these different effects on the biology of early HSC, we investigated how the t(9;22) fusion proteins interfere with the regulators of “stemness”, such as Wnt-signaling, c-Myc, HoxB4, Cdkn1a, Bmi-1 and SCL [19]. More specifically, we investigated the effect of the ABL/BCRs on Wnt-signaling by measuring Tcf/Lef transcriptional activity through determination Topflash/Fopflash reporter activity in U937 cells expressing BCR, p185*^BCR/ABL^*, p40*^ABL/BCR^* or p96*^ABL/BCR^*. U937 cells expressing PML/RARα, which is known to activate Wnt signaling, were used as a positive control. In these cells, BCR, p185*^BCR/ABL^* and p40*^ABL/BCR^* had no effect on Tcf/Lef-dependent transcription, whereas p96*^ABL/BCR^* activated Tcf/Lef-dependent transcription to a larger extent than did PML/RARα (Figure 2G). Next, we assessed the expression of HoxB4, Cdkn1a (p21^Cip1/Waf1^), c-Myc and SCL in Sca1^+^/lin^−^ HSCs expressing p185*^BCR/ABL^*, p40*^ABL/BCR^* or p96*^ABL/BCR^* by quantitative real time PCR (qRT-PCR). Figure 2H shows that, in contrast to p185*^BCR/ABL^*, both p40*^ABL/BCR^* and p96*^ABL/BCR^* induced the transcription of HoxB4. Conversely, p185*^BCR/ABL^*, but not p40*^ABL/BCR^* or 96*^ABL/BCR^*, up-regulated Cdkn1a expression. All of the fusion proteins down-regulated c-Myc, whereas only p185*^BCR/ABL^* and p40*^ABL/BCR^*, but not p96*^ABL/BCR^*, down-regulated SCL expression. Bmi-1 was not influenced by the t(9;22) fusion proteins (data not shown).

These findings indicate that all of the fusion proteins regulated the expression of genes involved in the maintenance of stem cell capacity; p185*^BCR/ABL^* up-regulated Cdkn1a and down-regulated c-Myc (Wilson et al. 2004), p40*^ABL/BCR^* up-regulated HoxB4 and down-regulated c-Myc and p96*^ABL/BCR^* activated Wnt-signaling, up-regulated HoxB4 and down-regulated c-Myc.

### All t(9;22)-associated fusion proteins increased the re-population potential of murine HSCs

**Figure 3:**
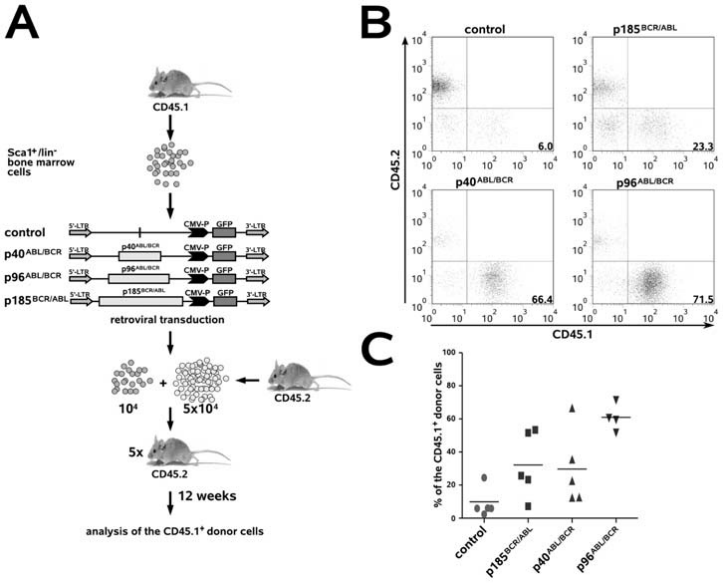
Effect of the t(9;22) fusion proteins on the re-population capacity of murine HSC. (A) The schematic indicates the experimental procedure for determination of the re-population potential of murine HSCs expressing t(9;22) fusion proteins. Sca1^+^/lin^−^ BM cells from CD45.1^+^ mice were infected with the indicated retroviruses and, at 48 h post-infection, the cells were transplanted together with CD45.2^+^ BM cells into lethally (11 Gy) irradiated CD45.2^+^ recipient mice. Analysis of donor chimerism was performed 12 weeks after transplantation. (B) Plots show representative donor-derived chimerisms from individual mice at 12 weeks post-transplantation. (C) Graph of donor-derived chimerism in each mouse after long-term reconstitution (4–5 mice/group).

To investigate whether and how p40*^ABL/BCR^*, p96*^ABL/BCR^* and p185*^BCR/ABL^* influence stem cell capacity, we performed a competitive repopulating assay (CRA). CD45.1^+^/Sca1^+^/lin^−^ HSCs transduced with p40*^ABL/BCR^*, p96*^ABL/BCR^* and p185*^BCR/ABL^* were inoculated together with CD45.2^+^ HSCs into lethally irradiated congenic CD45.2^+^ recipient mice. Chimerism was measured after 12 weeks in order to determine the re-population capacity of these cells as an index of their stem cell capacity (Figure 3A). Both ABL/BCR fusion proteins increased re-population; this effect was most pronounced with p96*^ABL/BCR^* (Figure 3B). Remarkably, p185*^BCR/ABL^* also conferred increased engraftment, although it did not increase serial replating efficiency, as shown above. Finally, at short time points after transplantation, the levels of chimerism in the peripheral blood were equivalent, suggesting that the observed effects were unlikely to be a consequence of differences in homing (data not shown).

Taken together, these data indicate that all t(9;22)-associated fusion proteins are able to increase stem cell re-population of Sca1^+^/lin^−^ HSCs.

### Both p40*^ABL/BCR^* and p96*^ABL/BCR^* have leukemogenic potential

**Figure 4:**
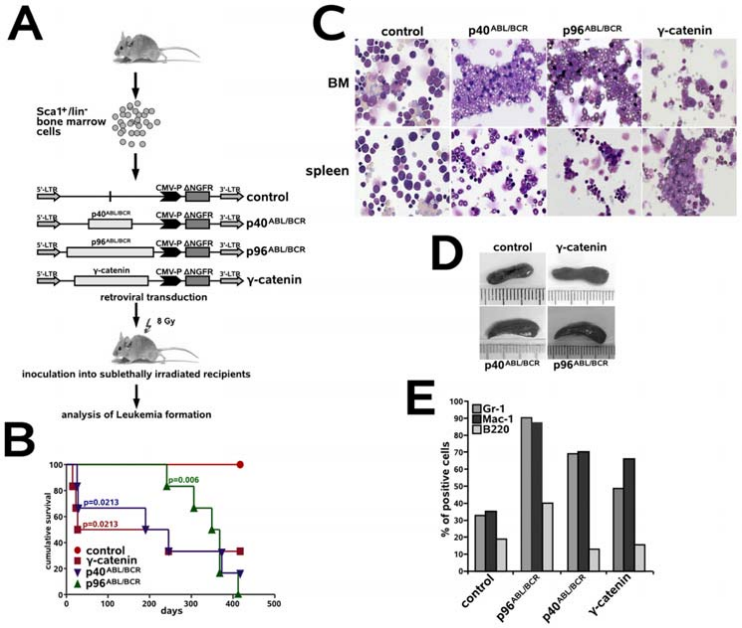
The leukemogenic potential of p40*^ABL/BCR^* and p96*^ABL/BCR^*. (A) Experimental strategy to model leukemia induced by the reciprocal t(9;22) fusion proteins. Sca1^+^/lin^−^ BM cells were infected with the indicated retroviruses and inoculated into sub-lethally irradiated mice. As positive and negative controls, we used γ-catenin and empty vector-transduced cells, respectively. (B) Survival curves show the frequency of recipients succumbing to disease after receiving the transduced cells. Statistical relevance was set at p<0.05. (C) May-Grünwald-Giemsa staining of cytospins from BM and spleen of one representative mouse in each group. (D) Relative splenomegaly of p40*^ABL/BCR^*-, p96*^ABL/BCR^*- or γ-catenin-positive leukemia. (E) Expression of differentiation-specific surface markers.

The fact that the ABL/BCRs induce a leukemic phenotype in HSC, which is characterized by a block of myeloid differentiation and increased stem cell capacity similar to that of other leukemia-associated fusion proteins [20], prompted us to investigate their leukemogenic potential. Thus, Sca1^+^/lin^−^ murine HSCs transduced with p40*^ABL/BCR^*, p96*^ABL/BCR^* and γ-catenin (positive control) were inoculated into sub-lethally (8 Gy) irradiated recipients. As controls, we used recipients inoculated with empty-vector transduced Sca1^+^/lin^−^ HSCs (Figure 4A). Mice inoculated with control γ-catenin-transduced cells became sick within 16–245 days, whereas p40*^ABL/BCR^* induced disease within 26–373 days after inoculation (Figure 4B). In contrast, mice inoculated with p96*^ABL/BCR^*-transduced cells showed a delay in the onset of disease (241–412 days). Penetrance for all transgenes was relevant (Figure 4B). The presence of both p40*^ABL/BCR^* and p96*^ABL/BCR^* induced a myeloid leukemia-like phenotype with maturation, associated with moderate splenomegaly (0.2–0.6 mg spleen weight)[21](Figure 4C–E). The WBC in the leukemic mice at diagnosis ranged from 15 to 34×10^3^ cells/µL (data not shown).

Taken together, these data show that both ABL/BCR fusion proteins are able to induce a leukemic phenotype *in vivo*.

### In contrast to p185*^BCR/ABL^*, p96*^ABL/BCR^* increases the serial replating potential of early stem cell derived B-lymphatic progenitors

**Figure 5:**
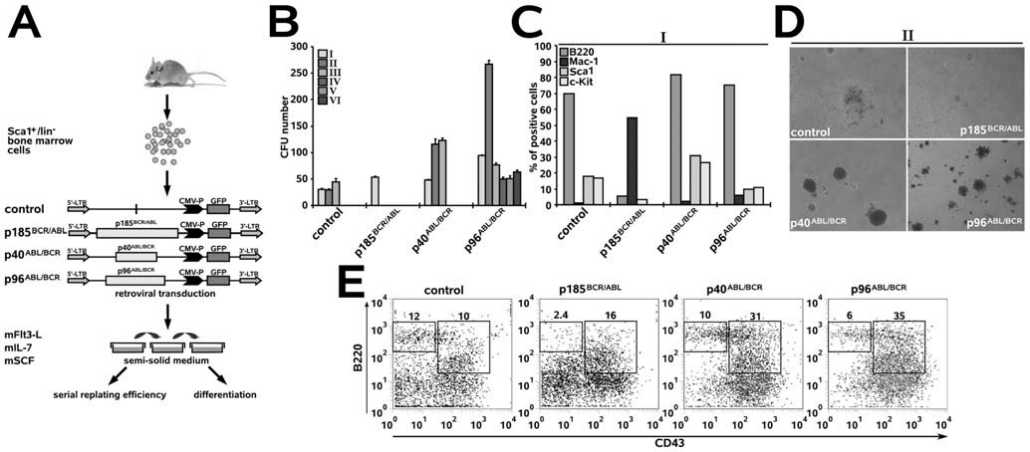
Differential effects of p40*^ABL/BCR^*, p96*^ABL/BCR^* and p185*^BCR/ABL^* on the B cell commitment of murine HSCs. (A) Experimental strategy for studying the influence of the reciprocal t(9;22) fusion proteins on the B cell commitment of murine HSCs. Sca1^+^/lin^−^ BM cells were infected with the indicated retroviruses and plated in semi-solid medium supplemented with the indicated growth factors for determination of the serial replating potential. Cells from the first plating (I) round and cells plated in liquid culture supplemented with the indicated growth factors were examined for the expression of differentiation-specific surface markers. (B) Long-term serial replating. Sca1^+^/lin^−^ cells were infected with the indicated retroviruses and plated into methyl-cellulose medium supplemented with the indicated growth factors to assess primary colony formation. Colony numbers were counted on days 8–10. Cells were then harvested and serially replated. Colonies were counted on days 8–10 for each replating. I-IV - number of the plating round. (C) Expression of differentiation-specific surface markers. (D) Colony morphology of the second plating. Type A (compact colonies), type B (a dense center surrounded by a halo of migrating cells) and type C (diffuse colonies with mobile differentiating cells) colonies were distinguished. p185*^BCR/ABL^* exhibited a high number of viable cells that were not organized into colonies. (E) Determination of the maturation stage of B220^+^ lymphocytes by CD43 expression analysis. CD43 positivity is characteristic of immature pro-B cells.

p185*^BCR/ABL^* and p96*^ABL/BCR^* are constantly and exclusively co-expressed in m-BCR-positive Ph^+^ ALL, which exhibits an immature phenotype blocked at an antigen-independent stage of differentiation. This prompted us to compare the effects of the ABL/BCR fusion proteins with those of p185*^BCR/ABL^* on the biology of HSC-derived B cell progenitors *in vitro*. Thus, the in vitro replating efficiency in semi-solid medium in the presence of SCF, Flt3 ligand (Flt3-L) and IL-7 of Sca1^+^/lin^−^ HSCs transduced with p40*^ABL/BCR^*, p96*^ABL/BCR^* or p185*^BCR/ABL^* was determined (Figure 5A). Here, we show that the replating efficiency of the empty-vector-transduced controls was limited to two cycles of replating (Figure 5B). In contrast, p96*^ABL/BCR^* increased the replating efficiency by over five cycles and also led to an increased number of CFUs. p40*^ABL/BCR^* augmented the number of CFUs, but not the replating efficiency as compared to the control cells, indicating an effect on proliferation (Figure 5B).

Both the p40*^ABL/BCR^*- and p96*^ABL/BCR^*-positive colonies exhibited a lymphatic phenotype as revealed by strong positivity for the B220 surface marker and absence of the Mac-1 marker for myeloid cells (Figure 5C). In contrast, the p185*^BCR/ABL^*-positive colonies were nearly completely negative for B220 and exhibited a myeloid phenotype as shown by positivity for Mac-1 (Figure 5C).

The morphology of the colonies was classified as type A, B or C according to the myeloid CFUs described above. As shown in Figure 5D, the expression of p40*^ABL/BCR^* led to compact type A colonies, whereas p96*^ABL/BCR^* gave rise to type A (about 40%) and smaller, less compact type B colonies (about 60%). Control cells exhibited the type B morphology with mobile cells. In contrast, p185*^BCR/ABL^*-positive cells did not exhibit colonies in the second plating round, but many viable cells were present that were apparently unable to give rise to colonies (Figure 5D). The expression of p40*^ABL/BCR^* and p96*^ABL/BCR^* induced a differentiation block as revealed by the fact that the majority of the p40*^ABL/BCR^*- and p96*^ABL/BCR^*-positive B220^+^ B cell population expressed CD43, a marker of the immature pro-B cell compartment. In contrast, most p185*^BCR/ABL^*-positive cells expressed the myeloid marker Mac-1, and the few lymphatic B220^+^ cells exhibited a more mature phenotype as revealed by the absence of CD43 expression (Figure 5E).

Taken together, these data suggest that, upon receipt of signaling that drives lymphatic commitment, p185*^BCR/ABL^* exhibits an apparently “paradox” phenotype characterized by expression of the myeloid surface marker Mac-1, while p40*^ABL/BCR^* increases proliferation and p96*^ABL/BCR^* fully transforms early B cell progenitors.

### BCR/ABL and p40*^ABL/BCR^* block, but p96*^ABL/BCR^* stimulates the generation of CD19^+^ B cell progenitors from human CD34^+^ UCBC

**Figure 6:**
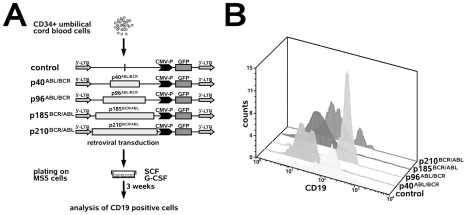
Differential effects of p40*^ABL/BCR^*, p96*^ABL/BCR^*, p185*^BCR/ABL^* and p210*^BCR/ABL^* on the B cell commitment of human HSC. (A) Experimental strategy for studying the influence of the reciprocal t(9;22) fusion proteins on the B cell commitment of human CD34^+^ UCBCs. CD34^+^ UCBCs were infected with the indicated retroviruses and plated on MS5 cells in the presence of SCF and G-CSF. After three weeks, the percentage of CD19^+^ cells was determined. (B) Histograms representing CD19^+^ cells in the GFP-positive cell population. The experiments were performed twice with similar results.

To exclude that the observed effects of the t(9;22) fusion proteins on B cell commitment were specific to murine HSC, we studied the effects of p185*^BCR/ABL^*, p96*^ABL/BCR^* and p40*^ABL/BCR^* on the generation of human B cell progenitors. Based on recent data showing that both p185*^BCR/ABL^* and p210*^BCR/ABL^* block B cell differentiation [22], we extended our experiments to the CML-associated p210*^BCR/ABL^* protein. To study early human B-lymphopoiesis, we used a co-culture system with a monolayer of the BM stroma cell line, MS-5, in which human CD34^+^ umbilical cord blood cells (UCBCs) differentiate to CD19^+^ cells within 3–5 weeks. The addition of SCF and G-CSF highly enhances the production of CD19^+^ cells [23]. Therefore, CD34^+^ UCBCs that were retrovirally transduced with p185*^BCR/ABL^*, p96*^ABL/BCR^* or p40*^ABL/BCR^* were co-cultured with MS5 stroma cells in the presence of SCF and G-CSF (Figure 6A). After three weeks, the percentage of CD19^+^ cells was determined. Herein, we show that the amount of CD19^+^ cells derived from CD34^+^ UCBCs expressing p40*^ABL/BCR^*, p185*^BCR/ABL^* or p210*^BCR/ABL^* was lower in comparison to that derived from p96*^ABL/BCR^*-expressing CD34^+^ UCBCs (Figure 6B).

In summary, these findings support our data on the influence of the t(9;22) fusion proteins on B cell commitment in the mouse.

### Opposite effects of BCR/ABL and ABL/BCR on key factors of lymphoid commitment and pre-B cell receptor signaling

**Figure 7:**
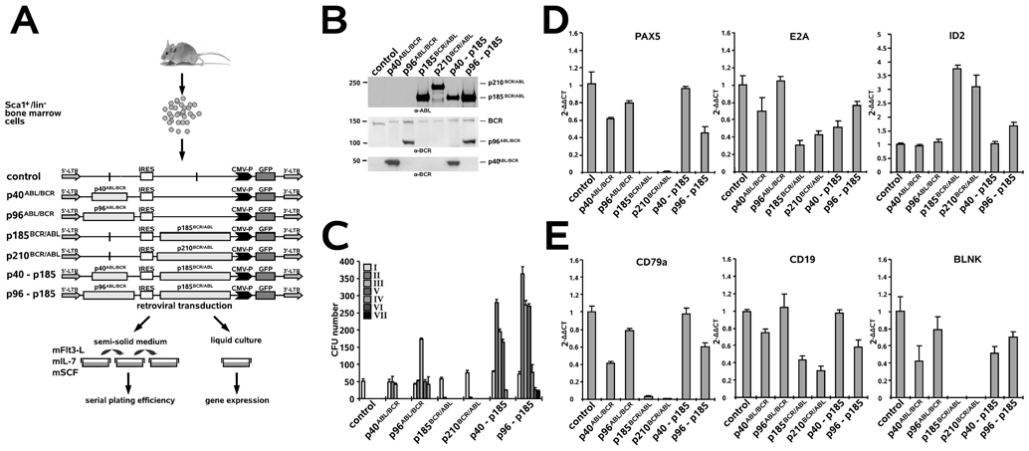
Influence of the t(9;22) fusion proteins alone or in combination on B cell commitment and expression of B cell-specific gene transcripts. (A) Experimental strategy for studying the influence of the reciprocal t(9;22) fusion proteins on the B cell commitment of murine HSCs. Sca1^+^/lin^−^ BM cells were infected with the indicated retroviruses and plated in semi-solid medium supplemented with the indicated growth factors for determination of the serial replating potential. (B) Transgene expression in transduced Sca1^+^/lin^−^ BM cells as determined by Western blotting using the indicated antibodies. (C) Long-term serial replating of Sca1^+^/lin^−^ cells infected with the indicated retroviruses and plated into wells containing methyl-cellulose medium supplemented with the indicated growth factors to assess primary colony formation. Colony numbers were determined on days 8–10. Cells were harvested and replated for the determination of the serial replating potential (D–E). Influence of the t(9;22) fusion proteins on key factors involved in B cell commitment and the pre-B cell receptor complex. Sca1^+^/lin^−^ BM cells were infected with the indicated retroviruses and, at 48 h post-infection, the expression of PAX5, E2A, ID2, BLNK, CD19 and CD79a were analyzed by qRT-PCR. The relative concentration of each mRNA was normalized to the concentration of the housekeeping gene GAPDH and is represented as 2^−Δ/Δ^ CT. Each experiment was performed in triplicate a total of three times with similar results. One representative experiment is given +/− SD.

Our data suggest that BCR/ABL and ABL/BCR exert different effects on the B cell commitment of early HSCs. Because ABL/BCR proteins are expressed together with the BCR/ABL fusion proteins in leukemic cells, we studied the influence of their co-expression with BCR/ABL on the biology of HSC-derived B cell progenitors *in vitro*. We retrovirally expressed p185*^BCR/ABL^*, p210*^BCR/ABL^*, p40*^ABL/BCR^* and p96*^ABL/BCR^* and the combinations of p185*^BCR/ABL^*/p40*^ABL/BCR^* and p185*^BCR/ABL^*/p96*^ABL/BCR^* in Sca1^+^/lin^−^ HSCs. These cells were plated either in semi-solid medium for the determination of their serial replating potential or in liquid culture for detection of the expression of key regulators of B cell differentiation; in both cases, the cells were cultured in the presence of SCF, Flt3-L and IL-7 as described in Figure 7A. The expression levels of the single transgenes did not differ between the cells that expressed the transgenes alone or in combination, suggesting that the observed effects were unlikely to be a consequence of differences in expression (Figure 7B). As shown in Figure 7C, neither p185*^BCR/ABL^* nor p210*^BCR/ABL^* increased the serial replating potential or the CFU number as compared to empty vector-transduced controls. Co-expression of p40*^ABL/BCR^* and p185*^BCR/ABL^* led to an increase in the CFU, indicating an effect on proliferation, whereas p96*^ABL/BCR^* together with p185*^BCR/ABL^* not only increased the CFU-number, but also the replating efficiency as compared to p40*^ABL/BCR^* and p96*^ABL/BCR^* alone. Taken together, these data suggest that the reciprocal t(9;22) fusion proteins cooperate in the determination of a leukemic phenotype for B cell progenitors.

The different phenotypic effects of p40*^ABL/BCR^* and p96*^ABL/BCR^* and the apparent “paradox” effect of p185*^BCR/ABL^* on B cell development prompted us to investigate the influence of the t(9;22) fusion proteins on fundamental steps of B cell development: commitment and pre-B cell differentiation. Here, we studied the influence of the t(9;22) fusion proteins on E2A and PAX5, which are both indispensable for the commitment of stem cells to the B cell lineage [24], [25]. Their expression was determined by qRT-PCR in Sca1^+^/lin^−^ HSCs expressing p185*^BCR/ABL^*, p210*^BCR/ABL^*, p40*^ABL/BCR^* or p96*^ABL/BCR^*, as well as those co-expressing p185*^BCR/ABL^* and p40*^ABL/BCR^* or p185*^BCR/ABL^* and p96*^ABL/BCR^*. As shown in Figure 7D, both BCR/ABL fusion proteins abolished the expression of PAX5 and strongly reduced that of E2A, in contrast to p40*^ABL/BCR^* and p96*^ABL/BCR^*, neither of which strongly impacted the expression of E2A or PAX5 alone. In cells co-expressing p185*^BCR/ABL^*, p40*^ABL/BCR^* almost completely reverted the abolition of PAX5, but not of E2A expression. Conversely, p96*^ABL/BCR^* only partially reverted the PAX5 suppression and nearly completely reverted the down-regulation of E2A. To understand how BCR/ABL regulates the expression of PAX5 and E2A, we determined the expression level of the transcription suppressor ID2, which is known to regulate both PAX5 and E2A [26], [27]. We found that both p185*^BCR/ABL^* and p210*^BCR/ABL^* up-regulated ID2, an effect that was nearly completely abolished by the ABL/BCRs, which did not have any effect on ID2 expression when expressed independently (Figure 7D).

The targeted disruption of structural components or down-stream signaling proteins of the pre-B cell-receptor complex results in the arrest of B cell development. Importantly, the expression and activity of proteins that are critical for pre-B cell receptor signaling are strictly regulated [28]. Therefore, we addressed the question of whether t(9;22) fusion proteins interfere with the expression of key components of the proximal pre-B cell signaling cascade, such as the adaptor protein BLNK (or SLP65)[29], the co-receptor CD19 and the signaling chain Igα (CD79a). As shown in Figure 7E, the BCR/ABLs abolished the expression of BLNK and CD79a and reduced that of CD19. The CML-associated p40*^ABL/BCR^*, in contrast to p96*^ABL/BCR^*, also reduced the expression of BLNK and CD79a, whereas neither of these proteins influenced the expression of CD19. It is noteworthy that both reverted, at least partially, the effects of p185*^BCR/ABL^* on the pre-B cell receptor complex components.

Taken together, these data strongly suggest that the BCR/ABL fusion proteins inhibit signaling pathways that are indispensable for normal B cell development at an early stem cell stage, an effect that is partially reverted by the reciprocal ABL/BCR fusion proteins.

## Discussion

Until now, the biology of Ph+ leukemia has been strictly limited to der22-encoded BCR/ABL and the related signal transduction pathways. Only CML-CP, a myeloproliferative syndrome, can be explained by the biology of BCR/ABL alone. It seems that p210*^BCR/ABL^* is able to induce and maintain indolent CML-CP. In fact, mathematical analysis together with studies on murine embryonic stem cells strongly suggests that BCR/ABL alone is sufficient to induce CML-CP without the need for any additional mutations [30], [31]. Progression to acute blast crisis is associated with additional mutations that might be independent of reciprocal ABL/BCR fusion protein expression, as suggested by the fact that p40*^ABL/BCR^* is not uniformly present in CML. In contrast, it is unlikely that an acute leukemia such as Ph+ALL is induced and maintained by only one mutation. The fact that p96*^ABL/BCR^* is uniformly expressed in Ph+ALL strongly suggests an important role for this fusion protein in the initiation and maintenance of Ph+ ALL.

Herein, we provide the first evidence that t(9;22) does not give rise only to one, but to two leukemogenic fusion proteins, the BCR/ABL and ABL/BCR fusion proteins. Both ABL/BCR fusion proteins are able to induce a leukemic phenotype *in vitro*, as characterized by a block of differentiation and, in the case of p96*^ABL/BCR^*, of an increased replating efficiency of primitive HSCs under conditions that permit myeloid commitment. In contrast, as already reported for p210*^BCR/ABL^* [32], p185*^BCR/ABL^* was not able to block differentiation of myeloid precursors or increase replating efficiency. The lack of increased replating efficiency can be explained by the fact that, in contrast to p40*^ABL/BCR^* and p96*^ABL/BCR^*, p185*^BCR/ABL^* up-regulated Cdkn1a expression and did not up-regulate Wnt-signaling or HoxB4 expression. The up-regulation of Cdkn1a together with the down-regulation of c-Myc likely contributes to the maintenance of stem cell capacity by p185*^BCR/ABL^*. These findings suggest that the influence of p185*^BCR/ABL^* on primitive HSCs is characterized by the induction of a “dormant” or “silent” state, whereas p40*^ABL/BCR^*- and p96*^ABL/BCR^* induce proliferation of HSCs. Hence, we hypothesize that the t(9;22) fusion proteins are active at different levels of the stem cell hierarchy [33].

Interestingly, p40*^ABL/BCR^* and p96*^ABL/BCR^* increased not only the stem cell capacity of primitive HSCs, but also exhibited leukemogenic potential, as shown in the transduction/transplantation model. The long latency, especially of p96*^ABL/BCR^*-positive leukemia, may be attributed to the need for additional mutations.

Previous data indicated that N-terminally truncated Rho-GEFs, such as NET1, may have oncogenic potential, mainly due to the presence of the Rho-GEF domain, which is defined by the DH- and PH-domains [15], [34]. Notwithstanding the differences between p40*^ABL/BCR^* and p96*^ABL/BCR^* regarding the presence of the putative oncogenic DH/PH domain, we have shown that both ABL/BCRs behave as hematopoietic oncogenes capable of inducing a leukemic phenotype *in vivo*. From our data, p96*^ABL/BCR^*seems to be the stronger oncogene as compared to p40*^ABL/BCR^* because of its capacity to increase the replating efficiency and stem cell capacity of primitive HSC. This characteristic might account for the aggressive biological behavior of Ph^+^ ALL compared to the initially indolent CP-CML.

The fact that p40*^ABL/BCR^* exhibits oncogenic potential may be attributed to its ability, which is shared by p96*^ABL/BCR^*, to activate Rac, a key player in the leukemogenesis of Ph+ leukemia. Furthermore, both ABL/BCRs inhibit Rho activation, which may contribute to the increase in stem cell capacity [11].

The activation of Rac is a common theme for ABL/BCR and BCR/ABL. This attribute may be the basis for the clinical relevance of the reciprocal t(9;22) fusion proteins, as they could maintain key signaling pathways, such as Rac signaling, upon effective inhibition of BCR/ABL kinase activity. In this way, the ABL/BCRs could play an important role in the grade of response to kinase inhibitors that target BCR/ABL. The capacity of ABL/BCR to autonomously induce leukemia suggests that the presence of p40*^ABL/BCR^* or p96*^ABL/BCR^* contributes to the maintenance of a leukemic subpopulation upon inhibition of BCR/ABL by ATP-analogues such as Imatinib, Dasatinib or Nilotinib, a scenario of particular importance in Ph+ ALL. One could speculate that the presence of ABL/BCR supports the proliferation of cells harboring BCR/ABL mutants with lower kinase activity, such as the T315I, which boasts nearly complete resistance to small molecules [35]–[36].

The clinical significance of p40*^ABL/BCR^* was addressed in several clinical studies that investigated the impact of the presence of der9 on clinical outcome; its significance is still controversially discussed [37]–[39]. A recent study on CML patients revealed that deletions of der9 spanning the ABL/BCR breakpoint, and thus abrogating the expression of p40*^ABL/BCR^*, were associated with an adverse prognosis [40]. Based on our data, one could speculate that these patients lacked the pro-proliferative effects of p40*^ABL/BCR^* at the stem cell level with a higher proportion of quiescent stem cells due to the up-regulation of Cdkn1a by BCR/ABL in HSCs [8].

In contrast to the BCR/ABLs, p40*^ABL/BCR^* and p96*^ABL/BCR^* allow for myeloid and lymphatic commitment of murine HSCs. These data are confirmed by our findings on the B cell development of human UCBCs with the exception of p40*^ABL/BCR^*blocks it like BCR/ABL. These different effects of p40*^ABL/BCR^* on murine and human HSC may be due to differences between UCBCs and murine HSCs regarding their “maturation” state.

Based on our data presented here, p185*^BCR/ABL^* and p210*^BCR/ABL^* do not allow the commitment of HSCs to the lymphoid lineage because, in stem cell-derived B cell progenitors, both of these proteins down-regulate PAX5 and E2A. Expression of both PAX5 and E2A is essential for B cell lineage commitment. The critical contribution of PAX5 to lineage selection is illustrated by loss-of-function studies. In its absence, pro-B cells assume a multipotential phenotype and differentiate (under appropriate growth factor conditions) to T cells, NK cells, dendritic cells, macrophages, neutrophils or erythroid precursors. Conditional PAX5 deletion results in the retrodifferentiation of B cells to an uncommitted progenitor cell stage [24]. In addition, E2A expression is essential for B cell lineage commitment. Similar to B cell progenitors that lack Pax5, E2A-deficient cells reconstitute T cell, natural killer cell, myeloid, dendritic and erythroid lineages, but not B cells, following transfer into lethally irradiated mice. Interestingly, similar to the reported multipotency of B cell progenitors that lack Pax5, E2A-deficient cells possess stem-cell-like properties, including the expression of genes that are normally associated with non-B cell lineages [25]. Therefore, the “paradox” behavior of p185*^BCR/ABL^*-expressing HSCs upon mIL-7, mSCF, and Flt3-L influence, which is indicated by the expression of myeloid surface markers, may produce a scenario similar to a PAX5 or E2A deletion with a blockage of B cell commitment. The mechanism by which BCR/ABL down-regulates PAX5 and E2A is most likely related to up-regulation of ID2. ID2 likely has other effects in addition to its ability to down-regulate PAX5. It is known that the DNA-binding activity of PAX5 is decreased in ID2-overexpressing B cells and enhanced in ID2^−/−^ B cells [26].

BCR/ABL not only inhibits key factors involved in B cell commitment, but also interferes with pre-B cell receptor signaling by down-regulating key proteins whose deregulation causes a block of differentiation at an early pre-B or pro-B cell level. This was confirmed by recent findings that indirectly showed an effect of BCR/ABL on pre-B cell receptor signaling. More specifically, it was shown that, in Ph^+^ ALL cell lines, expression of the main pre-B cell receptor signaling components was restored upon exposure to Imatinib [41].

The negative influence of BCR/ABL on B cell commitment strongly suggests that, for the determination of the Ph^+^ ALL phenotype, an additional mechanism that allows t(9;22)-positive precursors to engage in B cell commitment, or at least to maintain B cell characteristics, must be present. Together with the fact that 100% of M-BCR Ph^+^ALL cells expressed the reciprocal ABL/BCR fusion proteins, our observation that the ABL/BCRs at least partially reversed the effects of BCR/ABL on B cell commitment indicates that they are the first candidates for this role. This result is in apparent contradiction with the transgenic mouse models of BCR/ABL-positive ALL [42], [43]. In both models, additional mutations that substitute for the role of ABL/BCR can not be excluded. In fact, the expression of BCR/ABL alone was shown to mainly induce a CML-like disease in mice [44].

Taken together, we show for the first time that t(9;22) gives rise to two fusion proteins with leukemogenic potential, BCR/ABL and ABL/BCR, and our data provide the first evidence that, in m-BCR-positive ALL, p96*^ABL/BCR^* cooperates with p185*^BCR/ABL^* in the determination of the leukemic phenotype.

## Materials and Methods

### Plasmids

All cDNAs encoding p185*^BCR/ABL^*, p210*^BCR/ABL^*, p40*^ABL/BCR^* and p96*^ABL/BCR^* were described previously [11], [38]. All retroviral expression vectors used in this study were based on the bi-cistronic vectors PINCO or PAULO, with or without an internal ribosomal entry site (IRES) to drive translation of the two transgenes. PINCO and PAULO were converted into Gateway®-destination vectors by the introduction of a Gateway® cassette according to the manufacturer's instructions (Invitrogen, Karlsruhe, Germany). All related inserts were available in the Gateway® entry-vector (pENTR1A) for recombination into the destination vectors by using the “LR-clonase” enzyme kit (Invitrogen) [38].

### Cell Lines, Cell Culture, Transfection and Western Blotting

All cell lines were obtained by the German Collection of Microorganisms and Cell Cultures (DSMZ). BV173, SD-1 and U937 cells were maintained in RPMI 1640 supplemented with 10% FCS (Invitrogen). TOM-1 cells were maintained in Iscove's Medium supplemented with 10% FCS and SupB15 in RPMI 1640 supplemented with 15% FCS. U937 cells were transduced with transgenes by electroporation using the Nucleofection technology according to the manufacturer's instructions (Amaxa, Heidelberg, Germany). The ecotropic Phoenix packaging cells were cultured in Dulbecco's modified Eagle medium (DMEM; Invitrogen) supplemented with 10% FCS and were transfected by the calcium-phosphate precipitation method according to widely established protocols.

Western blot analyses were performed according to widely established protocols. The following antibodies were used: anti-ABL (α-ABL) (St. Cruz Biotechnology, Santa Cruz, CA, USA) and anti-BCR (α-BCR)(St. Cruz Biotechnology). Blocking and antibody incubation were performed in 5% low-fat dry milk, followed by washing in Tris-buffered saline (TBS) (10 mM Tris-HCl pH 8, 150 mM NaCl) containing 0.1% Tween20 (TBS-T).

### Topflash/Fopflash transactivation

The reporter constructs pRT-LK, pGL3-OT and pGL3-OF were electroporated by nucleofection into U937 cells together with isomolar quantities of the pCDNA3 expression vector encoding the transgenes of interest. After 24–36 h, luciferase activity was determined using the Dual-Luciferase Reporter Assay System according to the manufacturer's instructions (Promega, Mannheim, Germany). Luciferase activity was normalized using a co-transfected construct that encodes Renilla luciferase under the control of a PGK-promoter. Results are given as the ratio between Topflash and Fopflash luciferase activity.

### Patient samples and umbilical cord blood cells (UCBC)

Samples of mononuclear BM cells were obtained from patients with CML blast crisis or Ph+ ALL at first diagnosis and were frozen after informed and written consent with the approval of the ethics committee of the Goethe-University of Frankfurt. Immediately after thawing, the cells were lysed in Laemmli buffer and submitted to Western blot analysis.consecutive transduction rounds, every 8 to 12 hours, were done. Human umbilical cord blood was kindly provided by the Cord Blood Bank subdivision of the New York Blood Bank from healthy full-term pregnancies with the approval of the institutional review board. Human CD34+ cells were selected and CD34^+^ UCBC were enriched by immunomagnetic selection as described previously [45] and pre-stimulated for 48 hours in Quality Biological serum-free (QBSF)-60 medium (Quality Biological, Gaithersburg, MD) supplemented with c-Kit ligand (100 ng/ml; Kirin, Gunma, Japan), Flt3 ligand (100 ng/lL; Imclone Systems, New York, NY) and thrombopoietin (100 ng/ml; Kirin) prior to use.

### Isolation of Sca1^+^/lin^−^ HSCs

Sca1^+^/lin^−^ HSCs were isolated from 8 to 12-week-old female C57BL/6N mice (Harlan Winkelmann GmbH, Borchen Germany) after euthanization by CO_2_-asphyxiation. BM was harvested from the femura and tibiae by flushing the bones with a syringe and 26-gauge needle. The cells were “lineage depleted” by labeling the cells with biotin-conjugated lineage panel antibodies against B220, CD3ε, Gr-1, Mac-1 and Ter-119 (BD/Pharmingen, San Diego, CA). Labeled cells were removed using streptavidin loaded “MACS” cell separation columns. Sca1^+^ cells were purified by immunomagnetic beads using the “MACS” cell separation columns according to the manufacturer's instructions (Myltenyi, Bergisch-Gladbach, Germany). Prior to further use, the purified cells were pre-stimulated for 2 days in medium containing either mIL-3 (20 ng/mL), mIL-6 (20 ng/mL) and mSCF (100 ng/mL) (Cell Concepts, Umkirch, Germany) for myeloid commitment or mIL-7 (20 ng/mL), Flt3-L (20 ng/mL) and mSCF (100 ng/mL) for lymphatic commitment.

### Retroviral infection

Ecotropic Phoenix packaging cells were transfected with retroviral vectors as described before. Retroviral supernatant was collected at days 2 and 3 after transfection. Target cells were plated onto retronectin-coated (Takara-Shuzo, Shiga, Japan) non-tissue culture-treated 24-well-plates and exposed to the retroviral supernatant for 3 hours at 37°C in the presence of 4 µg/mL polybrene (Sigma, Steinheim, Germany). Cells were centrifuged at 2.200 rpm for 45 min. The infection was repeated 4 times and the minimal accepted infection efficiency was 70%, as assessed by the detection of GFP positive cells by Fluorescence-Activated Cell Sorting (FACS). Differences in the infection efficiency between samples did not exceed 10%.

For the retroviral transduction of human UCBC, vesicular stomatitis virus glycoprotein (VSV-G)-pseudotyped retrovirus was generated by transiently transfecting H29 cells with 10 ug of vector DNA by calcium phosphate precipitation. After 72 hours, the H29 supernatant was used for cross-transduction of PG13 packaging cells in the presence of polybrene (8 ug/mL; Sigma, St. Louis, MO). High-titer retrovirus-producing PG13 subclones were selected by cell sorting for high EGFP expression. Retrovirus-containing supernatants were harvested from PG13 clones incubated in QBSF for 8 to 12 h, supplemented with 100 ng/ml of c-Kit ligand, Flt3 ligand, thrombopoietin and 4 ug/ml polybrene, filtered through a 0.45-Am filter (Costar, Acton, MA), and applied immediately to the CD34^+^ cells on retronectin-coated six-well plates (Takara). Three consecutive rounds of transduction were performed.

### Cell cycle analysis

Sca1^+^/lin^−^ cells were collected from the cultures at 2 days post-infection, washed in PBS and fixed with 70% ethanol at −20°C. The cells were re-suspended in PBS containing propidium iodide (PI)(50 ug/ml) and RNAse (5 ug/ml) (Sigma, Steinheim, Germany) and incubated at 37°C for 30 min. The cells were immediately evaluated by FACS.

### Colony assays for determination of the colony forming cell (CFC) number and replating efficiency

At day 5 post-infection, Sca1^+^/lin^−^ cells were plated at 5×10^3^ cells/mL in methyl-cellulose supplemented with either mIL-3 (20 ng/mL), mIL-6 (20 ng/mL) and mSCF (100 ng/mL) for myeloid commitment or mIL-7 (20 ng/mL), Flt3-L (20 ng/mL) and mSCF (100 ng/mL) for lymphatic commitment (StemCell Technologies, Vancouver, Canada). On day 10 after plating, the number of colony forming units (CFU) was determined. After washing out from the methyl-cellulose, the cells were stained with specific antibodies for the detection of surface marker expression by FACS. 5×10^3^ cells/plate were replated in methyl-cellulose, thus permitting determination of the serial replating potency. Differentiation was assessed by the expression of c-Kit, Sca1, Gr-1 and Mac-1 (BD/Pharmingen) by FACS.

### Competitive re-population assay (CRA)

All animals studies were conducted in accordance with national animal protection laws and were approved by the Institutional Review Board (Regierungspräsidium Darmstadt - F 39/06). The CRA was based on the CD45.1 - CD45.2 chimerism of C57BL/6N mice as described previously (Bug et al., 2004). Two days after retroviral infection, 10^4^ CD45.1^+^/Sca1^+^/lin^−^ cells were injected into lethally (11 Gy) irradiated CD45.2^+^ female recipients together with 5×10^4^ normal Ly5.2^+^ BM cells. The portion of CD45.1^+^ donor cell-derived hematopoietic cells was determined by FACS analysis of cells after 12 weeks on BM – MNC. The cells were stained with conjugated monoclonal antibodies specific for CD45.1 and CD45.2 or with the mouse IgG2a isotype control (BD/Pharmingen).

### Transduction/transplantation model of leukemia

Female C57BL/6N mice that were 8–12 weeks of age (Harlan Winckelmann, Borchen, Germany) were used as recipients and donors. Sca1^+^/lin^−^ were isolated as described above. Recipients were sub-lethally irradiated with 8.5 Gy. 5×10^4^ transduced Sca1^+^/lin^−^ were inoculated into anesthetized mice by retro-orbital injection. The mice were sacrificed at the first appearance of morbidity, loss of weight (>10%), neurological abnormalities, failure to thrive or diarrhea. Statistical relevance was determined by the Log-rank test. The isolation of BM cells was performed as described above. Spleen cells were isolated by passing the tissue through a 40 µM Nylon cell strainer (Beckton-Dickinson, Le Pont de Claix, France). Whole BM and spleen cells were then cytospun on glass slides and stained with May-Grünwald-Giemsa. For surface marker analysis, the MNC were enriched on a Ficoll density gradient. Statistical relevance was determined by the Log-rank test.

### UCBC-derived B-lymphocytes

For B cell induction, 2×10^4^ transduced CD34^+^ cells were plated on MS-5 cells in MEM alpha supplemented with 10% FCS, 0.1 mM monothiolglycerol, 2 mM glutamine, 10 ng/ml huSCF and 5 ng/ml huG-CSF and were demi-depopulated on a weekly basis [23]. After three weeks, the suspension cells were harvested for further analysis.

### Quantitative real time PCR (qRT-PCR) – Taqman™

Total RNA and first strand cDNA were obtained from Sca1^+^/lin^−^ HSCs according to standard protocols. The TaqMan™ PCR was conducted in duplicate following standard protocols using the ABI PRISM 7700 (Applied Biosystems, Darmstadt, Germany). For the quantification of mRNA, gene expression quantification using “Assay-on-demand” for Cdkn1a, HoxB4, c-Myc, SCL, PAX5, E2A, ID2, BLNK, CD79 and D19 was performed according to the manufacturer's instructions (Applied Biosystems). Normalization to glyceraldehyde-3-phosphate dehydrogenase (GAPDH) was done for each sample. CT values were exported into a Microsoft Excel worksheet for calculation of fold changes according to the Comparative CT method. The amount of target, normalized to endogenous GAPDH, is given by 2^−Δ/Δ^ CT.
